# Spatial and temporal changes in gut microbiota composition of farmed Asian seabass (*Lates calcarifer*) in different aquaculture settings

**DOI:** 10.1128/spectrum.01989-24

**Published:** 2025-03-14

**Authors:** Melissa Soh, Shuan Er, Adrian Low, Zeehan Jaafar, Richard de Boucher, Henning Seedorf

**Affiliations:** 1 Temasek Life Sciences Laboratory 68741 , Singapore, Singapore; 2 Department of Biological Sciences, National University of Singapore 145755 , Singapore, Singapore; University of Mississippi, University, Mississippi, USA

**Keywords:** fish gut microbiota, aquaculture, Asian seabass, fish microbiome, microbial ecology, fish pathogens

## Abstract

The microbiota composition of healthy farmed fishes remains poorly characterized for many species. This study explores the influence of the external environment and innate factors that may shape the gut microbiota of farmed Asian seabass, *Lates calcarifer*. The α-diversity based on Shannon, Simpson, and Chao1 indices was lower for fishes reared in sea cages and tanks than for fishes that experienced a transfer from sea cages to tanks. Longitudinal analyses of gut segments revealed no significant differences in alpha diversity between segments within the same containment type, except for the Chao1 index between the stomach and pyloric cecum of sea-caged fishes. β-diversity analysis using weighted UniFrac distance and Bray-Curtis dissimilarity demonstrated that fish reared in the same containment type shared similar microbial communities. PERMANOVA tests confirmed that containment type, farm, and batch significantly influenced these distances. Containment type accounted for 10.4% of the observed diversity, farm for 29.8%, and batch for 10.7%. Genera comprising potential pathogens such as *Aeromonas*, *Flavobacterium*, and *Vibrio* were differentially abundant along the guts of fish from different containment types and particularly increased in tanks. Microbiota changes were observed with host age and gut segment, with differentially abundant microbial genera identified along the gut and as the seabass grew. Comparing the hindgut microbiota of Asian seabass to other species of farmed fishes revealed host-specific clustering as indicated by PERMANOVA. Overall, these findings underscore the significance of containment conditions on the gut microbiota of Asian seabass, with broad implications for aquaculture practices.

**IMPORTANCE:**

Understanding the microbiota composition of healthy farmed fishes is crucial for optimizing aquaculture practices. This study highlights the significant influence of containment conditions on the gut microbiota of farmed Asian seabass (*Lates calcarifer*). By demonstrating that gut microbiota diversity and community composition are shaped by containment type, farm location, and batch, the research provides valuable insights into how external environmental factors and innate host factors interact to influence fish health. The findings, particularly the differential abundance of potential pathogens in various containment types, underscore the need for tailored management strategies in aquaculture. This research not only advances our knowledge of fish microbiota but also has broad implications for improving the sustainability and productivity of aquaculture practices.

## INTRODUCTION

Cultured fish are an increasingly important food staple for human populations around the world. Disease outbreaks in aquaculture facilities therefore have a high economic impact while affecting food security (1). Previous studies on the microbial ecology of aquacultured fishes focused on disease outbreaks and the identities of pathogens in question (2). However, the microbiota of non-diseased fish species that are farmed remain poorly characterized. Closing this knowledge gap can influence farming practices as fish gut microbiota are important in host digestion, metabolism, stress response, reproduction, development, and pathogen infection (3–15). One possible mechanism for these observations could be the changes in metabolic processes catalyzed by the microbiome when different feed is offered (16).

We analyzed the gut microbiota of Asian seabass (*Lates calcarifer*), a species that is commonly farmed throughout the Indo-Pacific region, including Singapore. Wild Asian seabass feeds on fishes and crustaceans (17), and farmed seabasses are thus fed low-value fishes, raising concerns about the sustainability and environmental impact of these practices (18).

Common microbes associated with diseases in *L. calcarifer*, namely drug-resistant *Escherichia coli*, drug-resistant *Vibrio parahaemolyticus*, *Vibrio* spp., *Vibrio harveyi*, *Streptococcus iniae*, and *Tenacibaculum maritimum* have been investigated (19–23). Farm practices have been identified to influence the abundance of drug-resistant pathogens in host fishes (22). A recent study investigating the gut microbiota of diseased and asymptomatic Asian Seabass with mixed bacterial infections and chronic enteritis revealed a higher abundance of pathogenic microbes in diseased fish and the presence of beneficial microbes in asymptomatic hosts (23).

Published studies on fish gut microbiota overwhelmingly focus on the hindgut section of the intestinal tract, while other segments are often neglected. The exclusion of some portions of the intestinal tracts in these analyses is problematic. Teleost fishes, the most speciose group of vertebrate organisms, exhibit varied life histories, ontogenetic development, and diverse morphological variations. For example, major gut segments of a typical fish include the stomach, pyloric cecum, and the fore-, mid-, and hindgut (Fig. S1A). However, investigations into the comparative gut anatomy of teleost fishes reveal that not all segments of the alimentary canal are present in every fish species (24, 25). Consequently, examining longitudinal changes in gut microbiota along the major segments of the gastrointestinal tract will provide insights into how host anatomy and physiology correlate with bacterial composition, thus enhancing our understanding of host-microbe interactions. Studying gut microbes throughout the whole gut, rather than just the hindgut, also offers a greater potential for discovering probiotics and identifying specific gut segments that could benefit from existing probiotics. Knowledge of the microbial composition along the gastrointestinal tract can further improve the efficacy of prebiotics by allowing them to be formulated to target specific gut segments, thereby harnessing the gut microbiota to confer benefits to the host.

In this study, the microbiota of Asian seabass at different ages and under various farming conditions were investigated. In addition, the gut microbiota composition of *L. calcarifer* was compared to that of other fish species that are farmed. This study comprehensively assesses the gut microbiota of the Asian seabass and critically contributes to the foundation for more targeted interventions aimed at the gut microbiota.

## MATERIALS AND METHODS

### Sampling locations and methods

A total of 104 cultured seabasses were obtained from seven farms at different locations within Singapore; ranging from 38.9 g to 5.5 kg, and 11.6 cm to 63 cm standard length (Fig. S2). These fishes were either reared exclusively in sea cages or tanks; or transferred from sea cages to tanks. Of the farmed seabass, 62 were considered to be plate-/market-size. These fish were defined by host weight between 400 g and 1 kg. The plate-sized fish were derived from six of the seven farms, where four farms were sampled once each, and the remaining two farms provided two batches of samples each. For comparison, five wild-caught seabass were included in this study, of which three were plate-sized. These fish were obtained from local fishermen at two locations. The fish weighed between 411.5 g and 1793.9 g and had standard lengths between 27.7 cm and 49 cm.

Three other farmed fish species were also included in this study—milkfish (*Chanos chanos*, *n* = 3), red snapper (*Lutjanus malabaricus*, *n* = 8), and pomfret (*Trachinotus sp*., *n* = 7). Milkfish weighed between 555.1 g and 633.9 g, red snappers weighed between 564.2 g and 1015.4 g, and pomfrets weighed between 460 g and 625.4 g. All milkfish individuals were reared in sea cages within the same farm. Red snappers were obtained from two farms, in which six individuals were reared in tanks at one farm, and the remaining two individuals were reared in sea cages at the second farm. Three pomfrets were obtained from two farms each, and one individual was obtained from a third farm—all individuals were kept in sea cages prior to harvest.

All fish were kept and transported in ice once obtained and stored in a −80°C freezer in the laboratory until dissection. The identities of all fishes were made based on morphology and confirmed by sequencing their COI gene (26).

### Fish gut delineation and sampling

Fishes were buried in ice immediately after collection and during transportation. Sterile dissecting implements were used to extract guts from host fishes. The intestines of all individuals of the Asian seabass were demarcated into five portions: (i) stomach, (ii) pyloric cecum, (iii) foregut, (iv) midgut, and (v) hindgut (Fig. S1A). Demarcation was based on previous publications involving fish gut microbiota of several fish species (25, 27–29). Gut contents were squeezed into sample tubes to reduce the amount of host DNA in the resultant DNA extracted. Since each Asian seabass has multiple finger-like pyloric cecum, the contents of the pyloric cecum were pooled together in one sample tube. For each Asian seabass, gut contents from each segment were placed individually in screw cap tubes, homogenized, and stored at −80°C until microbial gDNA extraction. Only the hindgut was sampled for all individuals of milkfishes, red snappers, and pomfrets; all other methods follow those above.

### Sample processing

Genomic DNA was processed following methods in our earlier publication (26). In brief, gDNA was extracted from gut samples using 0.1 mm diameter zirconia beads with a bead-beating phenol-chloroform extraction method (30). Extracted gDNA was quantified using Quant-it Picogreen (Thermo Fisher Scientific, OR, USA), after which equal amounts of DNA were used in each PCR reaction. The V4 region of the 16S rRNA gene was amplified in triplicates using single index PCR with 515F and 806R primers, as described in the 16S Illumina Amplicon Protocol from the Earth Microbiome Project (31–33). A template-free negative control was included for each single index primer pair. We included the ABRF-MGRG 6 Strain Even Mix Genomic Material (ATCC MSA-3000) mock microbial community as a sequencing control. Each PCR contained the following: 30 ng gDNA template, forward and reverse primers at a final concentration of 0.2 µM each, 0.4U New England BioLabs Q5 High-Fidelity DNA polymerase, 5 µL 5× New England BioLabs Q5 buffer, the final concentration of 80 µM of Promega U1515 dNTP mix per nucleotide type and topped up to 25 µL using sterile molecular grade water. PCR was conducted using Bio-Rad thermal cyclers under the following conditions: 94°C for 3 min, 34 cycles of 94°C for 45 s, 50°C for 60 s, 72°C for 1:30 min, and elongation at 72°C for 10 min. PCR product triplicates were pooled and analyzed on 2% agarose gel. Based on DNA band intensity, the pooled triplicates were combined and then purified using AMPure XP (Beckman Coulter Genomics, Danvers, MA, USA). DNA quantity and quality were measured using Qubit and Nanodrop. PCR products were subsequently sequenced on the Illumina MiSeq platform using a 151 bp paired-end sequencing chemistry at the Genome Institute of Singapore.

### Sequence processing and microbiota analysis

Sequencing results were also processed following methods in our earlier publication (26). To process demultiplexed fastq files using QIIME 2 2021.4 (34), forward reads were first imported with the “qiime tools import” command, using options “--type SampleData[SequencesWithQuality]” and “--input-format SingleEndFastqManifestPhred33.” Each sequencing run was processed individually, where reads were denoised, dereplicated, chimera filtered, and trimmed to equal lengths using the command “qiime dada2 denoise-single,” with options “--p-trim-left 12” and “--p-trunc-len 150” (35). Feature tables were merged using the command “qiime feature-table merge” and option “--p-overlap-method sum,” while representative sequences were merged using “qiime feature-table merge-seqs.” “qiime feature-classifier classify-sklearn” and SILVA 138 SSU 99% identity 16S 515F/806R region database were employed to assign ASV taxonomic identity (36). The resultant feature table, together with “qiime feature-table filter-seqs,” was used to filter representative sequences. These representative sequences were then aligned using the command “qiime phylogeny align-to-tree-mafft-fasttree.” Alpha rarefaction curves were generated using “qiime diversity alpha-rarefaction” at “--p-max-depth 20,000,” and visualized at https://view.qiime2.org/ (37). Based on alpha rarefaction curves, rarefaction depth was set to be 4,029 since it provided sufficient sequencing depth. A rarefied feature table and diversity metrics were generated using the command “qiime diversity core-metrics-phylogenetic.”

### Overview of statistical methods

An ASV table indicating read counts of each ASV in each sample and a taxonomy table indicating microbial taxonomic classification for each ASV were obtained from QIIME2. The two tables, together with metadata describing each sample, were imported into R for statistical analyses and visualization (38). Sequencing results were processed using phyloseq, and ggplot2 was used for visualizations (39, 40). Natural logarithm was used for all logarithm calculations.

### Statistical analysis: relative abundance, ANCOM-BC, NMDS, PERMANOVA

Relative abundance of microbes was depicted at phylum and genus levels. In stacked barplots illustrating phyla relative abundance, the R package genefilter was used to filter phyla with relative abundance less than 0.5% each in all groups of samples to be labeled as “Others” (41). The Food and Agriculture Organization (FAO) collated a list of major diseases and the respective infectious agents that Asian seabass are vulnerable to (42). In the list, bacterial infectious agents were identified to the genus or species level. Of the bacterial pathogenic species, some shared the same genus. We thus collated a list of genera that were listed as an infectious agent or contained potentially pathogenic species, namely *Vibrio*, *Aeromonas*, *Pseudomonas*, *Streptococcus*, *Flavobacterium*, *Tenacibaculum*, *Cytophaga*, and *Chlamydia*. Since *Photobacterium damselae* subsp. *damselae* is considered an emerging marine fish and human pathogen, *Photobacterium* was also included in the list of selected genera (43, 44). Stacked bar plots were then generated to depict the relative abundance of the selected genera. Microbial composition was also analyzed with regard to core microbes. Core microbiota analysis was conducted using the core_members function from the R microbiome package, and default thresholds were used (detection threshold: 1%, prevalence threshold: 50%) (45).

To obtain differentially abundant taxa, ANCOM-BC was calculated using the ancombc2 function from the ANCOMBC package (46). The analysis was performed at the microbial genus level, and all other settings were set at default. The formula was set to consider segment, containment type, and host length in cm. When analyzing differentially abundant taxa among segments, hindgut samples were set as the reference group. In NMDS plots, points, centroids, and ellipses were generated at ASV resolution using vegan and BiodiversityR packages (47, 48). To calculate fitted vectors representing microbial taxa, ASVs were aggregated to phylum level before analysis. PERMANOVA was conducted using the adonis2 function from the vegan R package (47). Base R and R packages were used to generate the network between ASVs and individual fish while the strip plots were generated in Python 3.9. Gut segment samples were grouped for each fish. ASV abundances were transformed to relative abundance per fish, and only ASVs with relative abundance greater than 0.5% were retained. The network was displayed using a Prefuse Force Directed Layout. Cytoscape 3.9.1 and R package Rcy3 were used in network generation (49, 50). For the strip plots, ASVs were first categorized into two groups—detected in fish from only one farm (unique) and detected in fish from more than one farm (shared). Mean relative abundances of ASVs were then determined for each group, following which the ASVs were sorted in descending order of mean relative abundance, and only the top 20 ASVs of each group were displayed in the strip plots.

## RESULTS

### Containment type influences α- and β-diversity of the gut microbiota of farmed Asian seabass

**Fig 1:**
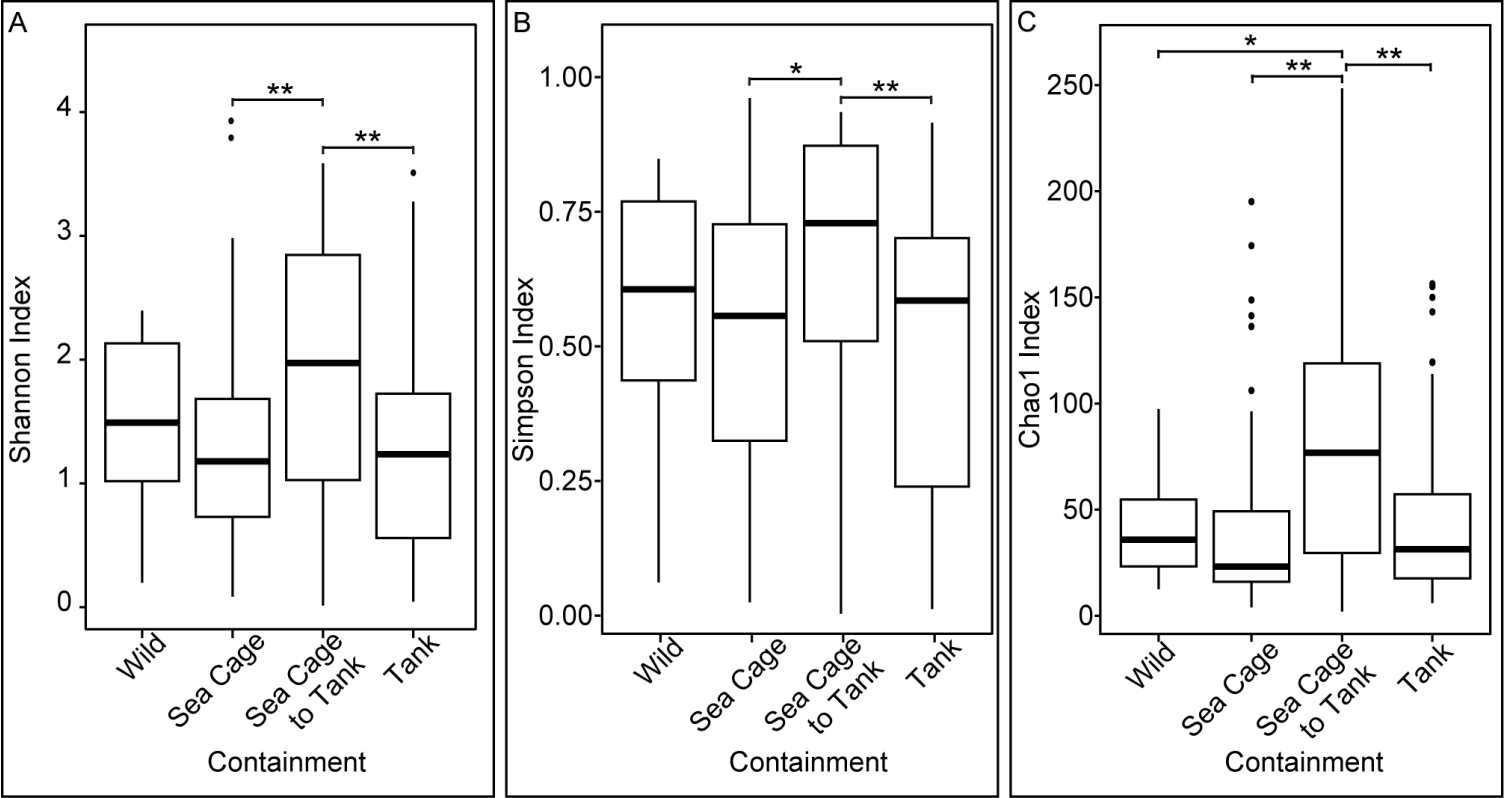
Contrasting gut microbial α-diversity of seabass from different containment types. Data derived from wild and plate-sized farm Asian seabass. (**A**) Shannon index, (**B**) Simpson index, and (C) Chao1 index of gut samples, where samples from the same containment types were grouped, regardless of segments. Fish transferred between containment types had the highest gut microbial α-diversity. Similar trends are observed across all three α-diversity indices used (*P*-values: *<0.05, **<0.01, ***<0.001).

Both environmental and innate factors may influence gut microbiota. To identify drivers of microbial α-diversity, gut samples of wild and marketable-sized farmed seabass were first grouped based on the containment the host was reared in. Fishes reared exclusively in sea cages or tanks had significantly lower Shannon (Fig. 1A), Simpson (Fig. 1B), and Chao1 (Fig. 1C) indices for gut microbiota than fishes that underwent containment type change (Wilcoxon rank-sum tests, Shannon index comparisons: both *P*-values 0.0055, Simpson index comparisons: sea cage vs transferred fish *P*-value 0.0181, tank vs transferred fish *P*-value 0.0097, Chao1 index comparisons: sea cage vs transferred fish *P*-value 0.0027, tank vs transferred fish *P*-value 0.0082). Concurrently, for Shannon and Simpson indices, there were no significant differences between wild and farmed fishes from all three containment types (*P*-value > 0.05). The Chao1 index of wild fish gut microbiota was significantly lower than that of fishes transferred between containment types (*P*-value 0.0356). Given the influence of containment type on α-diversity, to investigate the effect of the gut segment on gut microbial diversity, samples were grouped by containment type followed by segment (Fig. S1B through D). No significant differences were observed between all segments within the same containment type for all three α-diversity indices used (Wilcoxon rank-sum test, all *P*-values > 0.05) except for the Chao1 index between the stomach and pyloric cecum of fishes reared in sea cages (*P*-value < 0.05).

**Fig 2:**
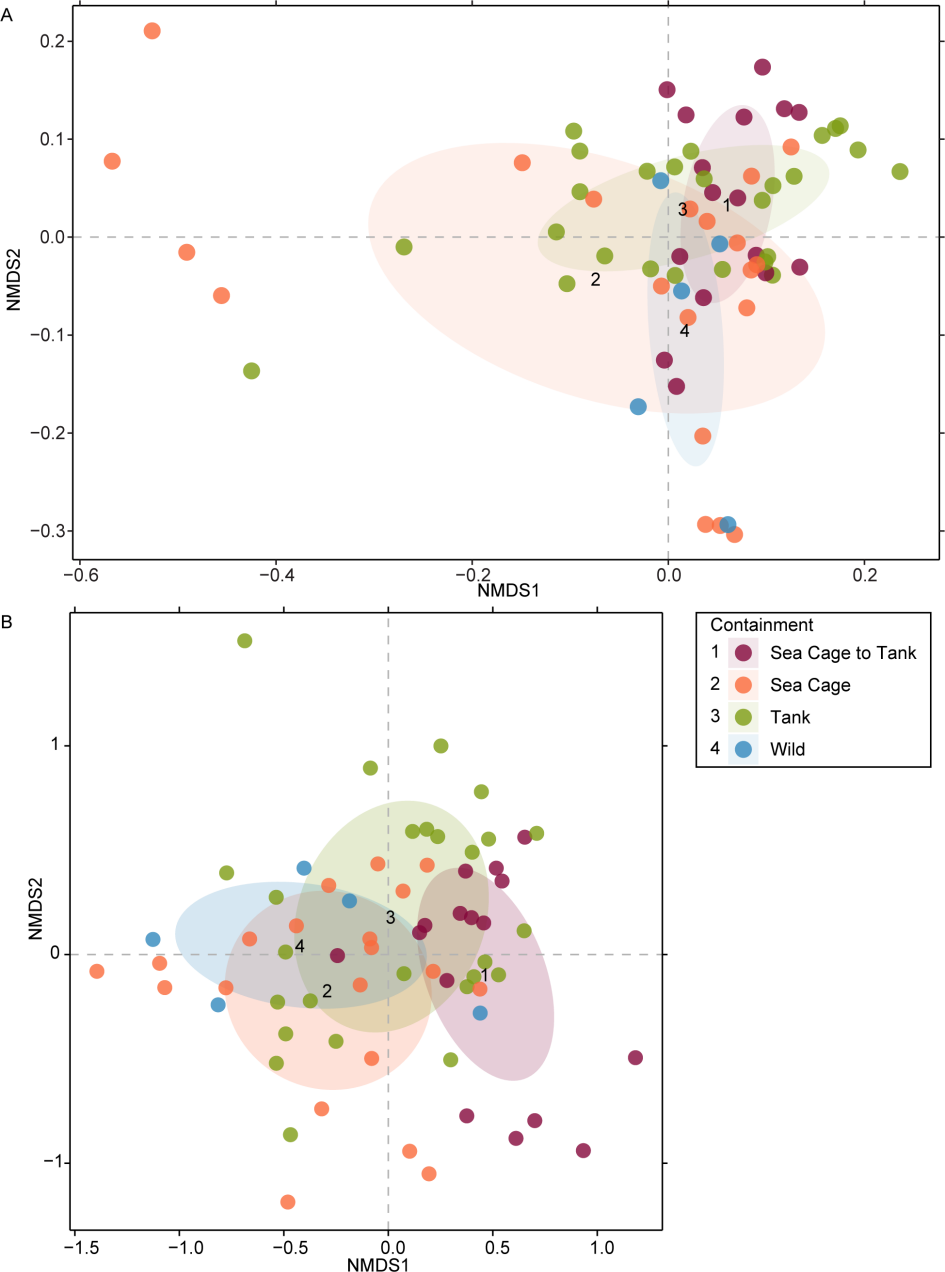
β-diversity of wild and plate-sized farm Asian seabass. NMDS was calculated using (A) weighted UniFrac distances and (B) Bray-Curtis dissimilarity. All segments from each fish were grouped at one point on the plot and colored based on the containment in which the fish were kept. Ellipses were similarly colored and generated to indicate standard deviation from centroid, with each centroid represented by a number.

Closer phylogenetic relationships between microbes potentially indicate metabolic similarity (51). As such, analyzing weighted UniFrac distances between samples can highlight the β-diversity difference both in terms of relative abundance and potential metabolic profile. Analyses of the effect of containment type on β-diversity, using both weighted UniFrac and Bray-Curtis dissimilarity, indicated that fishes reared in the same containment type are more similar (Fig. 2). PERMANOVA tests on the same data set revealed that containment type significantly influenced weighted UniFrac distances (*P*-value = 0.002, *R*^2^ = 0.1235) and Bray-Curtis dissimilarity (*P*-value = 0.001, *R*^2^ = 0.1220).

**Fig 3:**
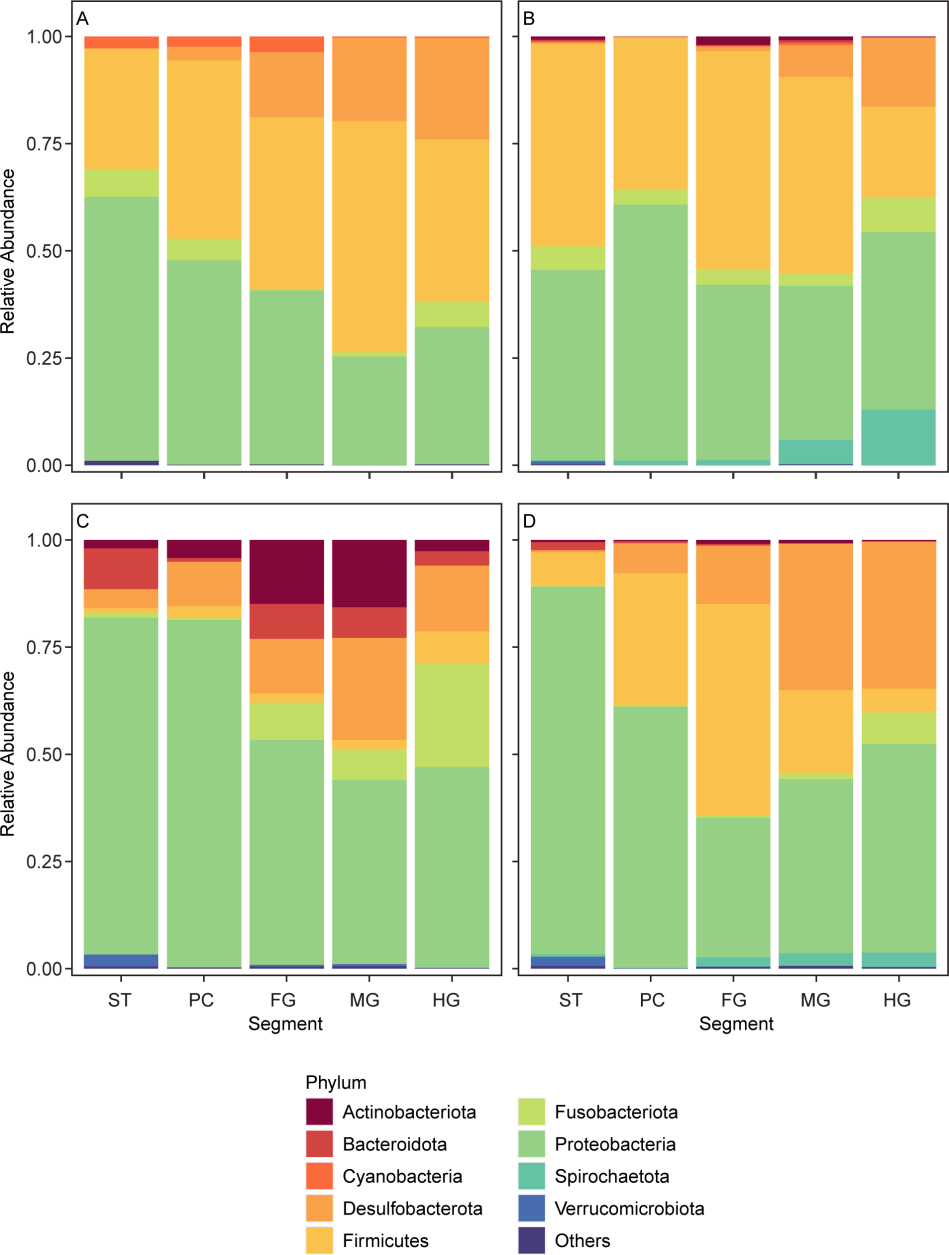
Distribution of microbial phyla along the gut length of wild and plate-sized farm seabass. Samples are grouped based on host containment. (**A**) Wild-caught seabass, (**B**) seabass kept in sea cages, (**C**) seabass transferred from sea cages to tanks, and (D) seabass kept in tanks.

Farm and batch effects have been found to influence gut microbiota in studies of other animal species such as rabbits and chickens (52, 53). As such, weighted UniFrac distances between samples were plotted and visualized based on farms and sampling batches (Fig. S3). For comparison, wild seabass samples were also included in marketable-sized farmed seabass weighted UniFrac distance calculations. Clustering patterns and ellipses indicate that farm and batch effects contribute to weighted UniFrac distances. To quantify the effect of cage type, farm, and batch effects on marketable-sized farmed seabass, PERMANOVA was conducted where the effects of containment type, farm, and sampling batch were sequentially calculated (R^2^ of containment type: 10.42%, farm: 29.82%, batch: 10.70%; all *P*-values = 0.001). Analyses based on Bray-Curtis revealed that samples from different farms and sampling batches cluster in close proximity (Fig. 3). PERMANOVA conducted on marketable-sized farmed seabass indicates that containment type accounted for 10.6% of Bray-Curtis dissimilarity, sampling farm accounted for 15.8% of the remaining diversity, and sampling batch accounted for 13.6% of the remaining diversity (all *P*-values = 0.001, terms tested sequentially).

**Fig 4:**
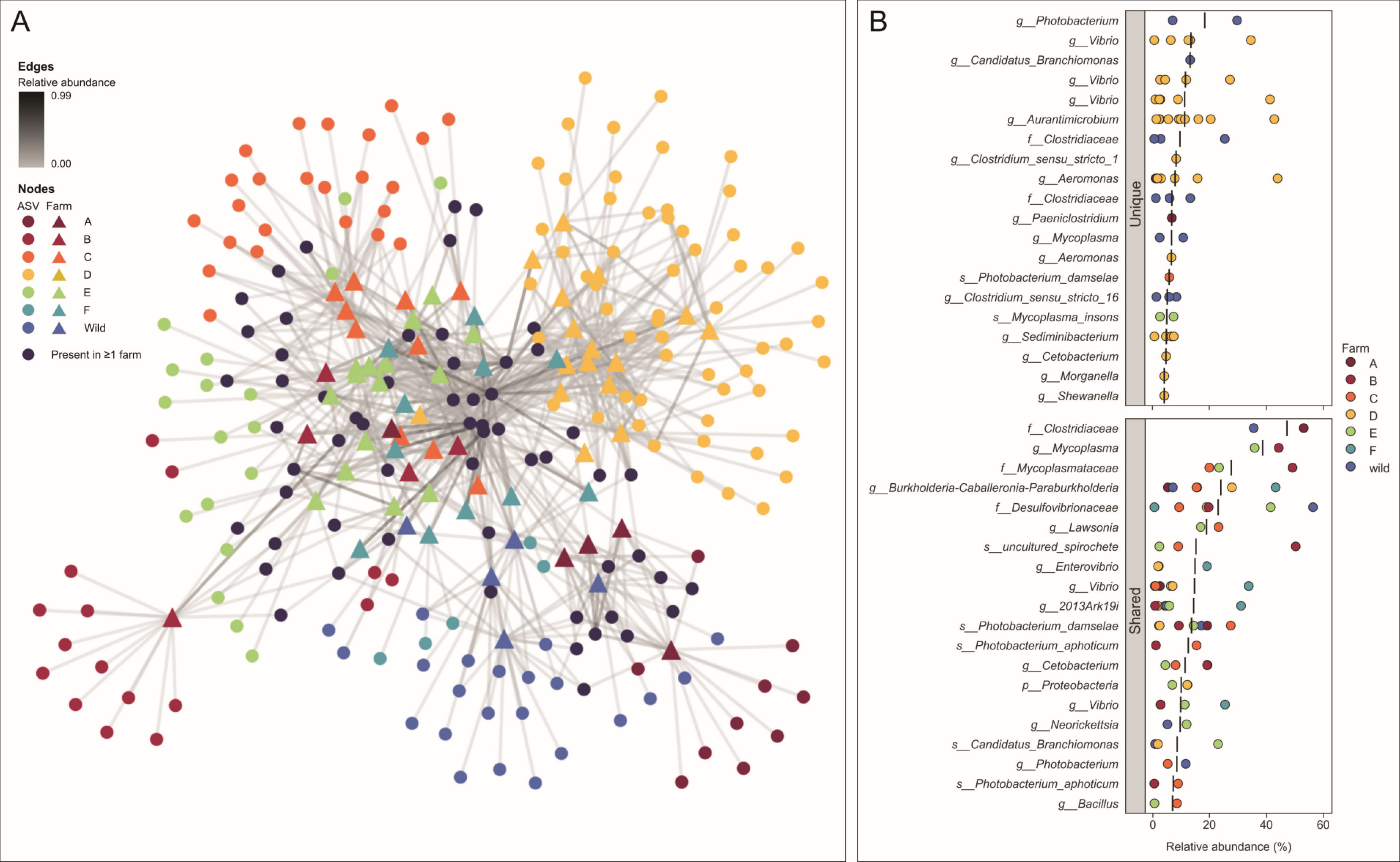
Overview of shared and unique bacteria associated with wild and plate-sized farmed seabass. (**A**) The network depicts the relationship between ASVs and fish where segments from each fish were grouped. ASVs that were found only in fish from one farm were colored based on where the fish was obtained. ASVs that were found on more than one farm were colored dark purple. (**B**) Dot plots illustrate the 20 most abundant ASVs found in only fish from one farm (unique) or detected in more than one farm (shared). Black vertical bars represent the mean relative abundance of the ASV.

In addition to α- and β-diversity, containment type influences gut microbiota at the phyla level (Fig. 3). Desulfobacterota showed increasing relative abundance along gut segments of fishes from all four containment types. Furthermore, Proteobacteria was a dominant phylum observed regardless of containment type (averages across all five segments: 41.5% in fishes harvested from the wild, 44.5% in sea cage reared fish, 60.4% in fishes transferred from sea cage to tanks, and 53.7% in fishes reared in tanks). Firmicutes was also dominant in fishes from three containment types (wild: 40.34%, sea cage: 40.16%, tank: 22.55%), with the exception being fishes transferred from sea cage to tank (3.13%). By contrast, Actinobacteriota was more abundant in fishes transferred from sea cages to tanks (7.87%) than fishes from the other three containment types (wild: 0.10%, sea cage: 0.79%, tank: 0.53%). In the former category, of the 85 Actinobacteriota ASVs, the most abundant ASVs were assigned to *Aurantimicrobium*, with no species designation. This ASV comprised 81.13% Actinobacteriota in transferred fishes. *Aurantimicrobium* was not observed in fish from the wild, sea cages, or tanks. Using a network analysis, it was observed that farm fish harbor ASVs that may be unique to a farm or ASVs that are shared by two or more farms (Fig. 4). It is noteworthy that both farm-unique ASVs and shared ASVs can be present at high relative abundance, which may indicate that some of the farming processes and/or environmental conditions may be unique to a farm, while others may be shared among them. Some of the highly abundant ASVs that are shared by at least two farms, Clostridiaceae, *Mycoplasma*, Mycoplasmataceae, *Burkholderia-Caballeronia-Paraburkholderia*, Desulfovibrionaceae can only be assigned to genus or family level, which may be due to the lack of cultured representatives of these particular clades.

### Prevalence of potential fish pathogens in Asian seabass gut

**Fig 5:**
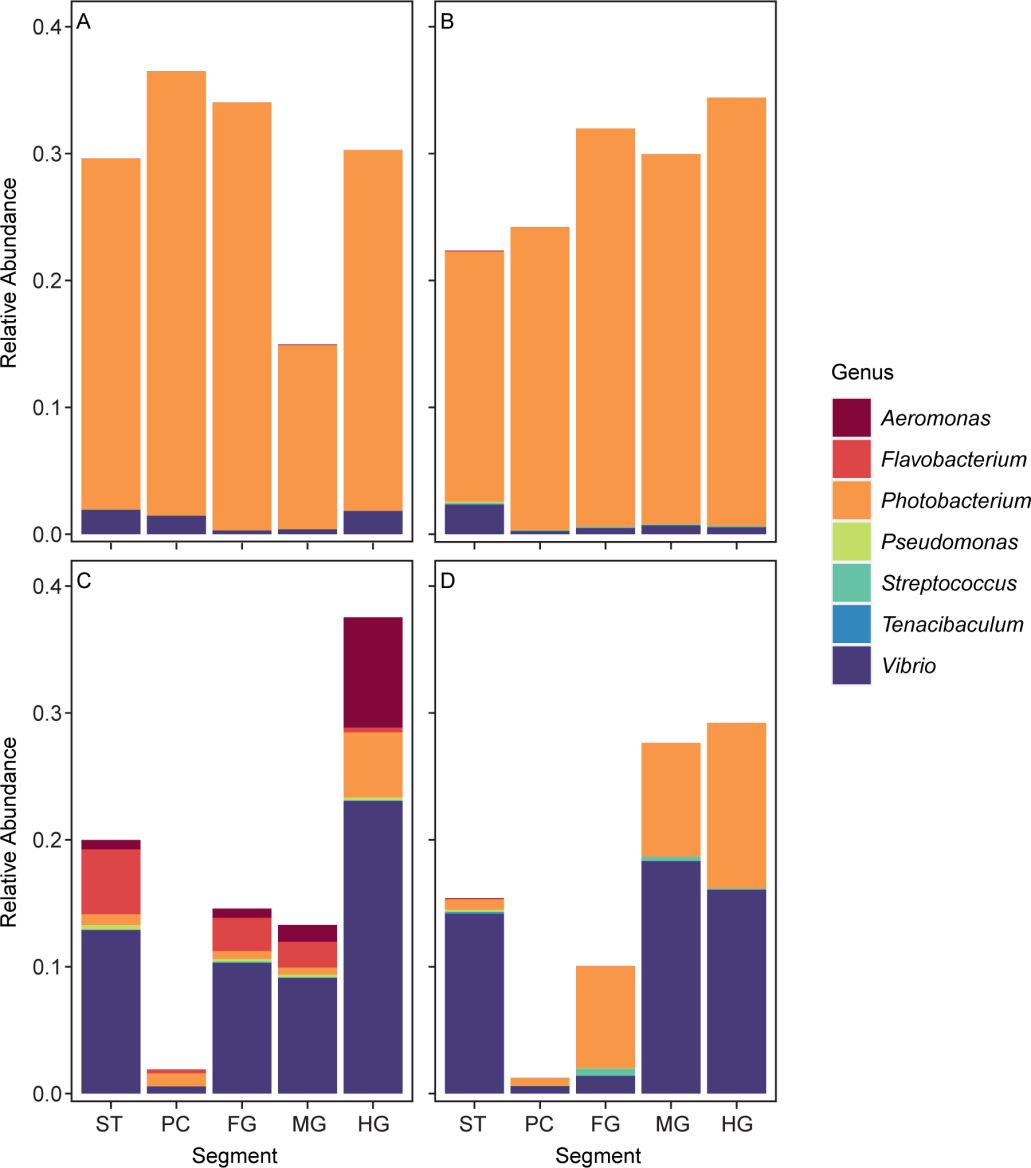
Relative abundance of selected microbial genera. In wild seabass and plate-sized farm seabass, genera composition varies along the length of the gut and depending on containment type. (**A**) Wild-caught seabass, (**B**) fish from sea cages, (**C**) fish transferred from sea cages to tanks, and (D) fish from tanks.

Disease outbreaks are a major concern in aquaculture facilities. Of the eight genera selected based on the FAO list of Asian seabass major diseases and their respective infectious agents (42), six genera were observed in the analyzed samples (*Aeromonas*, *Flavobacterium*, *Pseudomonas*, *Streptococcus*, *Tenacibaculum*, and *Vibrio*). In addition, *Photobacterium* was also observed. These seven genera were differentially abundant along the guts of fishes reared in different containments (Fig. 5). The distribution of the selected genera varied between fishes from different conditions. For instance, wild fishes and fishes reared in sea cages had high relative abundance of *Photobacterium* (wild: 27.9%, sea cage: 27.6%, transferred from sea cage to tank: 1.6%, tank: 6.3%), while fishes transferred between sea cages and tanks, and fishes reared in tanks, had higher relative abundance of *Vibrio* (wild: 1.2%, sea cage: 0.9%, sea cage to tank: 11.2%, tank: 10.1%). *Aeromonas* was only present in fish transferred between sea cages and tanks (2.3%).

### Longitudinal changes in microbiota along the major gut segments and fish age

**Fig 6:**
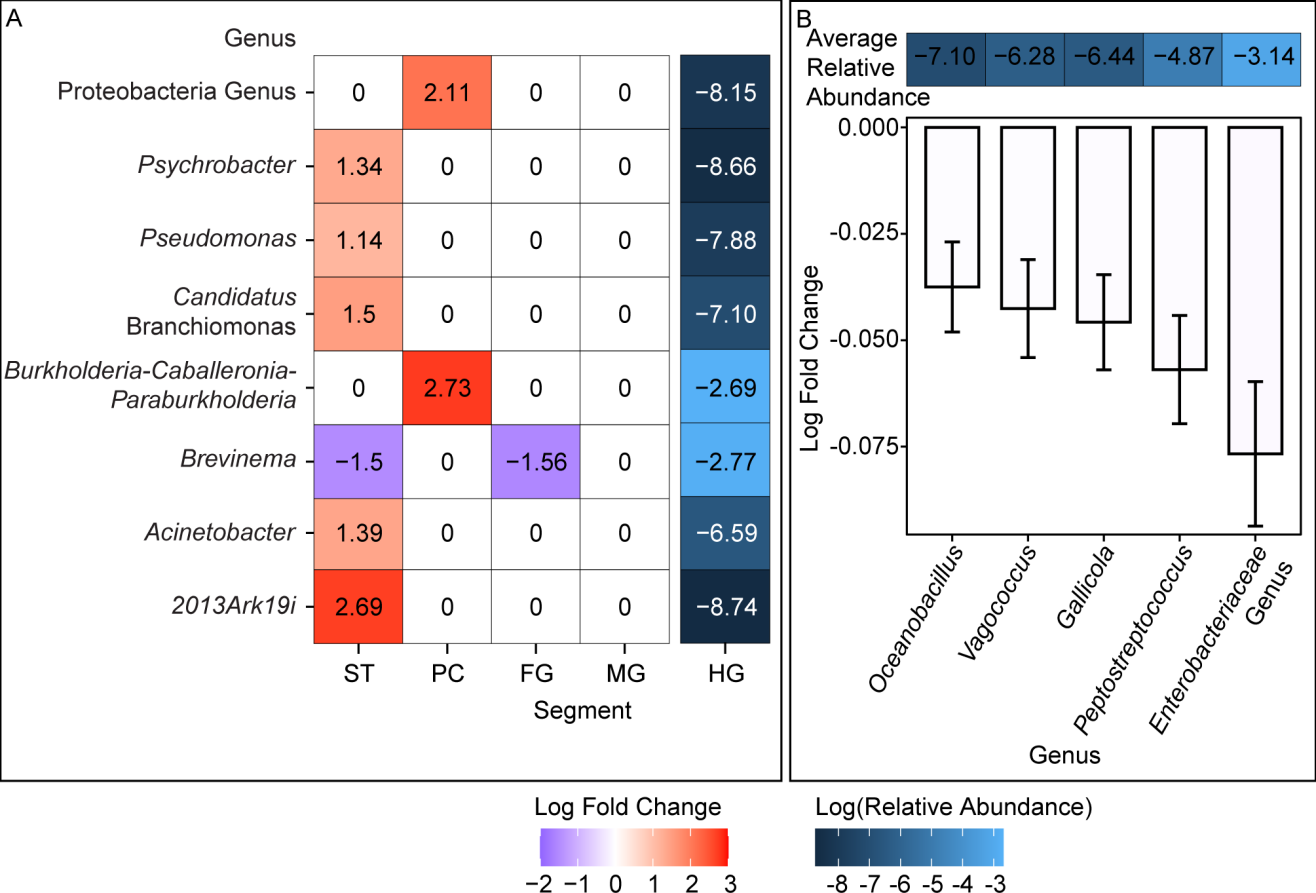
Spatially and temporally differentially abundant microbial taxa. All farm seabass samples were used in this analysis. Taxa grouped by genus, or next available taxa when a genus could not be assigned. LFC and relative abundance are depicted using different color scales. Natural logarithm was used for all logarithm calculations. Possible relative abundance ranges from zero to one, with one representing 100% relative abundance. (**A**) LFC of microbial genera along the length of the gut from stomach to midgut. The relative abundance of the genera in the hindgut, which was used as the reference segment, was also illustrated. (ST: stomach, PC: pyloric cecum, FG: foregut, MG: midgut, HG: hindgut) (B) LFC of microbial taxa with increasing host standard length. Average relative abundance illustrated on top of the panel.

In many species, gut microbiota changes along the gut, possibly due to changes in the physical environment within the gut lumen (54–56). An NMDS analysis based on Bray-Curtis dissimilarity did not reveal obvious visual trends regarding samples or farming practices (Fig. S4). However, differentially abundant microbial genera were identified longitudinally between major seabass gut segments (Fig. 6A). When using the hindgut as a reference, the number of differentially abundant taxa decreased along the gut. More specifically, except for *Brevinema*, all differentially abundant taxa observed in the stomach and pyloric caecum had a higher log-fold change (LFC) than compared to those in the hindgut. Since host length is commonly used as a proxy for host age (57, 58), to understand changes in microbial composition as the host aged, differentially abundant microbial genera were also identified as the seabass grew (Fig. 6B). All differentially abundant taxa decreased in LFC as host length increased.

PERMANOVA was conducted using Bray-Curtis dissimilarity on farm seabasses of various lengths. The marginal effects of intrinsic parameters (“host length,” “weight,” and “gut segment”) and extrinsic parameters (“containment type”) revealed that “gut segment” was responsible for the highest percentage of Bray-Curtis dissimilarity among samples (R^2^ values for “host length”: 2.30%, “weight”: 1.70%, “gut segment”: 5.15%, “containment type”: 4.83%; all *P*-values = 0.001). Results of PERMANOVA conducted using weighted UniFrac were similar (R^2^ values for “host length”: 2.19%, “weight”: 2.53%, “gut segment”: 5.57%, “containment type”: 5.32%; all *P*-values = 0.001).

### Similar gut microbiotas among farmed fish species

**Fig 7:**
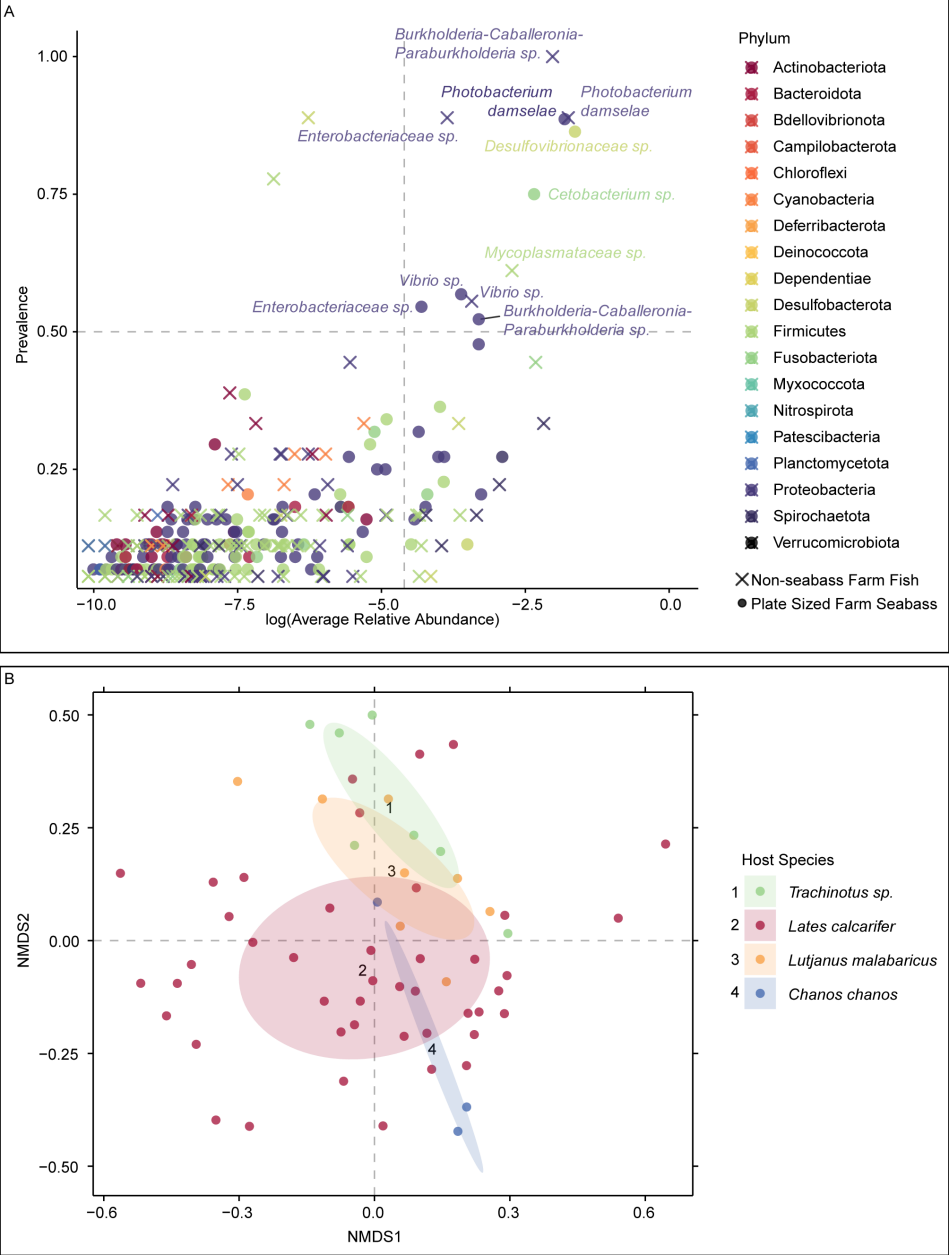
Comparing gut microbial diversity of aquaculture fish of different species. Gut microbiota of hindgut samples from plate-sized seabass and non-seabass fish species, *Trachinotus* sp., *L. malabaricus*, and *C. chanos*, were included. (**A**) Relative abundance and prevalence of ASVs where dots represent ASVs observed in seabass, while crosses represent ASVs observed in non-seabass fish species. Gray dashed lines demarcate the threshold for core microbiota at the detection threshold 1% and a prevalence threshold 50%. Core ASVs are labeled with the lowest taxonomy classification available. Samples above the 50th percentile were illustrated in this plot. (**B**) NMDS depicting Bray-Curtis dissimilarities between gut microbiota of the four farm fish species. Ellipses indicate standard deviation from the centroid, which is indicated by numbers.

To investigate host specificity of Asian seabass gut-associated microbiota, we compared the hindgut microbiota of Asian seabasses to those of three species of farmed fishes, *Trachinotus* sp., *Lutjanus malabaricus*, and *Chanos chanos* (Fig. 7A). Out of the 690 ASVs found in the Asian seabass hindguts and 360 in the non-seabass fish hindguts, only several ASVs were of sufficient relative abundance and prevalence to be considered core ASVs. More specifically, there were two core ASVs uniquely present in marketable-sized farmed Asian seabass hindguts (*Cetobacterium* sp. and *Desulfovibrionaceae* sp.), one core ASV uniquely present in non-seabass farmed fish hindguts (*Mycoplasmataceae* sp.), and four core ASVs shared between Asian seabass and non-seabass hindguts (*Enterobacteriaceae* sp., *Vibrio* sp., *Burkholderia-Caballeronia-Paraburkholderia* sp., and *Photobacterium damselae*). The four shared core ASVs had a total average abundance of 35.84% in non-seabass and 23.94% in seabass guts. We also investigated the β-diversity of gut microbiota of the four species of farmed fishes (Fig. 7B). In general, there was host-specific clustering of the samples from the different fish species. Using the same data set, PERMANOVA indicated that there was host specificity in the microbiota diversity of the samples. Fish species accounted for 14.11% of the β-diversity differences (*P*-value = 0.001). When taking farm and batch into consideration, fish species accounted for 8.49% (*P*-value = 0.001) (“farm and batch” R^2^ = 26.24%, *P*-value = 0.001).

## DISCUSSION

This study comprehensively characterized the gut microbiota of both wild and farmed healthy Asian seabasses at different weights, age ranges, and containment types. Since this study considered multiple parameters, the results elucidated the microbial constituents of Asian seabass guts and the drivers of their diversity. Focusing on one farmed species allowed deep investigations into host-specific parameters, in addition to other factors such as containment types. Samples from wild-caught individuals of Asian seabass were also included in several analyses to contextualize the impact of containment, and containment types, on gut microbiota diversity.

### Influence of containment type on gut microbiota

The gut microbiota of fishes transferred between containment types had higher α-diversity and gross compositional differences, especially at the phylum and genus levels compared to fishes of other containment types. Fishes subjected to regular transfer between containments may have been stressed, which has been shown to change the gut microbiota in a wide range of animals, including fishes. In a study on Atlantic salmon, chronic environmental stresses and acute cold stresses were related to an increased abundance of Gammaproteobacteria, in particular *Acinetobacter* and *Aeromonas* (59). Cortisol may be a potential mediator of stress-induced microbiota alterations. Juvenile Atlantic salmon exposed to stress via confinement had increased concentrations of fecal cortisol, which were, in turn, positively correlated with the Shannon index of fecal microbiota and an increased abundance of Gammaproteobacteria operational taxonomic units (60).

Bacterial diseases such as Flavobacteriosis, Tenacibaculosis, and Vibriosis are common in Asian seabass (61). In this study, there are striking similarities between the relative abundance of potential fish pathogens between wild and sea cage fishes. The close proximity between wild fish and sea cage fishes may increase the transfer of microbes, including pathogens (62). Wild fishes are attracted to sea-based farms likely due to the structural and food availabilities afforded by the sea cages and feeding activities of captured fishes (63). While not all species of the selected genera of microbiota have been identified as pathogens, the presence of closely related taxa may promote host gut colonization by the invading species (64). In both the wild and sea cage fishes, *Photobacterium* was the dominant genus among those investigated. In a previous study, *Photobacterium damselae* subsp. *damselae* was isolated from both wild and cage-cultured marine fish, where the isolates were found to harbor antibiotic-resistance genes (62). Other studies also recorded the antibiotic resistance of *Photobacterium damselae*, possibly indicating that the microbial genetic similarity between wild and cage-cultured fishes warrants further investigation (65, 66). The disparate spatial distribution of *Flavobacterium* spp. found mainly in Asian seabass individuals transferred from sea cages to tanks can be further investigated. The result indicated that *Flavobacterium* was not shed in high enough abundance in the hindgut compared to the upper gastrointestinal tract, which impacts the viability of using fecal samples as a non-invasive method of detecting *Flavobacterium* as studied elsewhere (67). Subsequent studies should focus on the detection of *Flavobacterium covae* and *Flavobacterium columnare*, known pathogens in diseased Asian seabasses (68).

Similarities between the gut microbiota of wild and sea cage fishes were also observed in β-diversity analyses. PERMANOVA results indicated that containment type partially accounted for gut microbiota β-diversity, and that farm and sampling batch explained some of the remaining diversity. Differences in farming practices between farms and changes in farming practices within farms over time might explain some of the farm and batch effects. These practices include feed formulation, antibiotic use, and starvation duration before harvest. Other reasons include variables outside of the control of the farm, such as seasonal changes in water parameters such as temperature and salinity. These variables have been found to influence gut microbiota in several fish species (69–73). Furthermore, captive fishes, especially those in enclosed settings, could be more affected by batch effects. Wild marine fishes experience a continuous flow of seawater which can remove waste materials, while captive fishes in tanks can experience volatile water conditions since rapid changes in water parameters have been linked to changes in feeding rate, water volume, and fish stocking density (74). The large sample size of this study, which included fishes of diverse lengths and from different farms, could ameliorate the impacts of undocumented specific farming practices on the study outcome. Furthermore, the emergence of generalized trends from this study signifies a robust sampling effort, especially when considering the variability between farms and batches. Specific characterization patterns of gut microbiota (e.g., microbial composition, α-, and β-diversity) can be elucidated from the spatial distribution of gut microbiota along the different gut segments as well as the temporal distribution of gut microbiota across fish ages. Finally, cage, farm, and batch effects emphasize the high sensitivity and responsiveness of gut microbiota and the need to contextualize information on gut microbiota to environmental parameters.

### Temporal and spatial variation in gut microbiota

In this study, PERMANOVA results revealed that among all farmed seabasses analyzed, the parameter “gut segment” was the driver for microbial β-diversity, compared to “containment type,” “host weight,” and “length.” Regarding the Asian seabass, an increased relative abundance of Desulfobacterota, sulfidogenic bacteria, was observed along the length of the gut. Sulfidogenic bacteria such as *Bilophila wadsworthia* have been implicated in the respiration of taurine, a critical amino acid in the fish diet, and is commonly conjugated with primary bile acids (75). In vertebrate organisms, bile is released from the gallbladder into the proximal gut (76). Only six out of 55 observed Desulfobacterota ASVs have cultured representatives. Two of the cultured ASVs were identified as *Pseudodesulfovibrio profundus*, and the rest were *Desulfovibrio alaskensis*, *Desulfovibrio capillatus*, *Desulfovibrio senezii*, and *Olavius algarvensis*. Additional characterization of Desulfobacterota might provide further insights into the functional role of the microorganisms in the Asian seabass gut. If a strong association between Desulfobacterota abundance and bile salts is established, monitoring Desulfobacterota abundance might allow clues on bile acid status in fish since the latter is often disrupted by fishmeal alternatives (76).

At the genus level, stomach microbiota had the highest number of differentially abundant genera, and almost all these genera are of higher abundance than in the reference hindgut segment. *Brevinema* was the only differentially abundant genus that was of lower abundance in the stomach than the hindgut. *Brevinema* has been found in the gut microbiota of several fish species (77–79). This genus is a spirochete and a known pathogen in humans (80, 81). Spirochetes also perform beneficial ecological roles by acetogenesis and dinitrogen fixation in termites (82). They have also been suggested to contribute to host protein metabolism in fishes, possibly explaining the increased abundance in the hindgut of Asian seabass (82–84). The only differentially abundant genus between foregut and hindgut was a reduced *Brevinema* abundance in the foregut. There were no other differentially abundant genera between fore-, mid-, and hindgut. This similarity between the three segments could be attributed to the lack of physical structures demarcating the segments. The physical structure of the pyloric cecum might provide insight into its function. The sac-like structure of the pyloric cecum is inconducive to continuous flow-through of digesta and is postulated to increase gut surface area for increased absorption (25, 85). The Asian seabass pyloric cecum was found to have two differentially abundant genera compared to the hindgut, namely increased abundance of an unclassified Proteobacteria genus and *Burkholderia-Caballeronia-Paraburkholderia*. Facultatively anaerobic Proteobacteria have been speculated to create an anaerobic environment in the gut by using oxygen and reducing environmental redox potential (86). At the same time, some species of *Burkholderia-Caballeronia-Paraburkholderia* can perform anaerobic metabolic processes (87, 88). For instance, *Burkholderia cepacia* NRRL B-18403 was reported to reduce aromatic compounds under anaerobic conditions using nitrate as an electron acceptor (89). Together, it is possible that on top of an increased surface area, the pyloric cecum also provides a suitable environment for microbes that would otherwise not thrive in the gut, allowing the breakdown of nutrients that would have remained undigested. In all, our study indicated that the gut microbiota of Asian seabasses varies spatially, across the gut segments. This emphasizes the importance of also considering a segment-based microbiota analysis. However, more analyses will be required to determine the physiological role of microbiota in the respective gut segment (and at different growth stages), especially when also considering that the amount of contents may vary significantly between segments.

The gut microbiota of Asian seabass also varies with age. Temporal variations in gut microbiota have been previously reported in other fish species where host gut microbiota diversity decreases with age (90, 91). In this study, as Asian seabasses grew, all the differentially abundant genera reduced in abundance. Of the five differentially abundant genera of Asian seabass ages, three (*Oceanobacillus*, *Vagococcus*, and *Peptostreptococcus*) have been reported to comprise some species that are potentially pathogenic to humans and/or other animal species (92–94). Since decreases in relative abundance do not necessarily translate to decreases in absolute abundance, future work investigating absolute abundance can provide insights into whether there is a decrease in absolute abundance as the host ages. A recent publication established a new method of estimating microbial biomass that could be useful (95). If there is a decrease in the absolute abundance of potential pathogens in the Asian seabass gut as the host grows, there are two implications. First, in commercial farms, most fish mortality occurs at the fry and juvenile life stages. To improve fish survival, more research could be conducted into potential pathogens that are more abundant in younger than older fishes. Second, the reduction in potentially pathogenic genera as host ages can be construed as positive, since it indicates a reduced exposure to these potential pathogens when preparing larger fish for consumption.

### High similarity between core gut microbiota of four farm fish species

Host-specific differences in the β-diversity of gut microbiota among the four species of farmed fishes were observed. When focusing on the core microbes, the four common core ASVs represented most of the core ASVs of both seabass and non-seabass-farmed fishes. This suggests that the core microbiota of fishes farmed within the marine waters of Singapore may potentially be location-specific and could have limited host specificity. Gut microbiota composition was suggested to be location specific, based on a study in China where the gut microbiota of eight species of farmed fishes were examined (96). The researchers investigated core microbes at the familial and generic levels and identified 11 taxa that were core members of the gut microbiota of the seven fish species. There were differences in the abundance of core microbes within a host species sampled from different regions of the coast, leading the authors to conclude that location influences the resultant fish gut core microbiota. The gut microbiota of five species of fishes reared in close proximity to each other in sea cages revealed the presence of the same seven operational taxonomic units, of common core microbiota in all host taxa (97).

Research involving more species of farmed fishes within the region is required to determine the presence of a core gut microbiota in fishes farmed in Singapore and to elucidate the site specificity of this core microbiota. Considering the possibility of site specificity, and not host species specificity, in determining the gut microbiota of farmed fishes, we hypothesize that the core microbiota provides functions for fish health that is important to the host for thriving in the specific environment. Since the functions are essential, fishes, regardless of species, have a common core microbiota. In this paper, ASVs were identified based on the V4 region of the 16S rRNA gene sequence. Microbial genome sequences could indicate that the shared core ASVs are potentially belonging to different microbial strains. To confirm whether there is host specificity in the core gut microbiota of farmed fish in Singapore, microbial genomes need to be obtained. Likewise, it will also be important to obtain more detailed information on farming practices, for example, the type of feed used, to obtain a more comprehensive picture of the factors that may shape the fish gut microbiota.

### Conclusion

This study investigated the gut microbiota of a wide size range of wild and farmed Asian seabasses. In general, there were spatial and temporal variations in Asian seabass gut microbiota. Extrinsic (containment type) and intrinsic (gut segment and age of host) parameters influence the gut microbiota differently. Microbial changes along gut segments revealed information on possible pyloric caecum function and the nitrogen cycle in the host gut. Some focus was placed on the distribution and abundance of select microbial genera, which can impact future aquaculture applications, such as targeting gut segments during treatment against potential pathogens or planning containment types. Lastly, there could be location specificity but limited host specificity in determining the gut core microbiota of fish farmed in Singapore. Expanding the analyses to other geographic regions as well as to metabolomic (and other “omics”) analysis may further improve our insights into the structure and function of the farmed Asian seabass microbiome.

## SUPPLEMENTAL MATERIAL

Supplemental material spectrum.01989-24-s0001.pdf Fig. S1 to S4.

ASM does not own the copyrights to Supplemental Material that may be linked to, or accessed through, an article. The authors have granted ASM a non-exclusive, world-wide license to publish the Supplemental Material files. Please contact the corresponding author directly for reuse.
