# Supplementary material for: Spatial and temporal changes in gut microbiota composition of farmed Asian seabass (Lates calcarifer) in different aquaculture settings
Source: Microbiol Spectr. 2025 Mar 14;13(5):e01989-24. doi: 10.1128/spectrum.01989-24 (PMC12054105; doi:10.1128/spectrum.01989-24)
Supplement: Supplemental material — Fig. S1 to S4. [file spectrum.01989-24-s0001.pdf]

Supplementary Figures

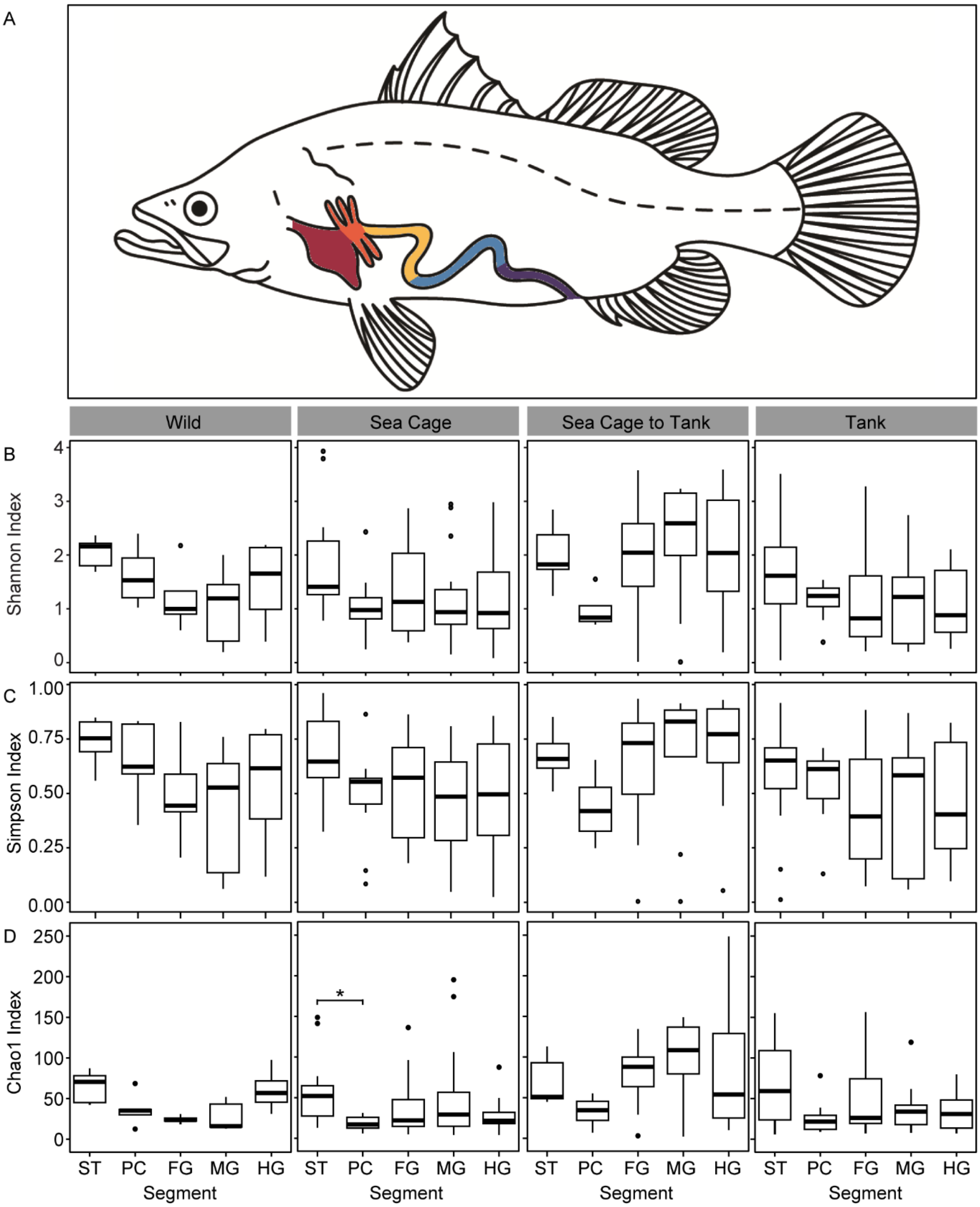

Supplementary Figure 1: Analyzing microbial diversity along the fish gut. A) Demarcation of Asian seabass gut. Seabass gut portioned into five segments: Stomach (red), pyloric cecum (orange), and three equal lengths of foregut (yellow), midgut (blue), and hindgut (purple). Gut

microbial α-diversity changes along length of gut, grouped by containment types, measured using three α-diversity indices, B) Shannon index, C) Simpson index, and D) Chao1 index. All p-values within cage type (between segments) are > 0.05, with the exception of Chao1 index of gut microbiota from stomach and pyloric cecum of fish reared in sea cages. (P-values: * <0.05, ** <0.01, *** <0.001)

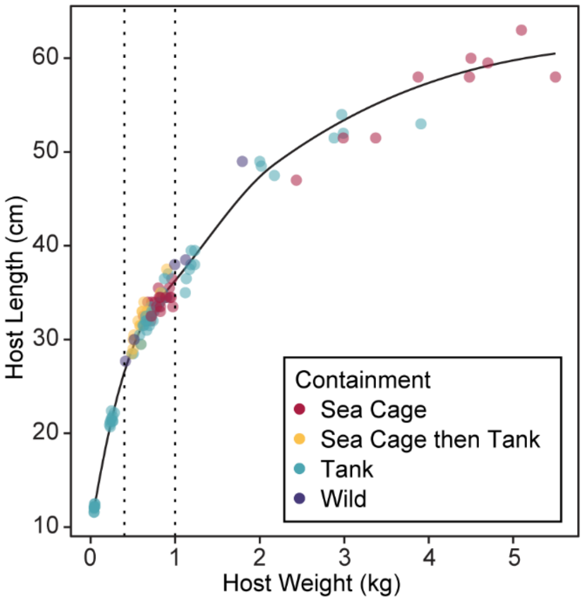

Supplementary Figure 2: Weights and lengths of wild and farm seabass sampled. Each point colored by containment the host was kept in. Black curve fitted using LOESS regression. Dotted lines demarcate weight range of plate-sized seabass, 400g to 1kg.

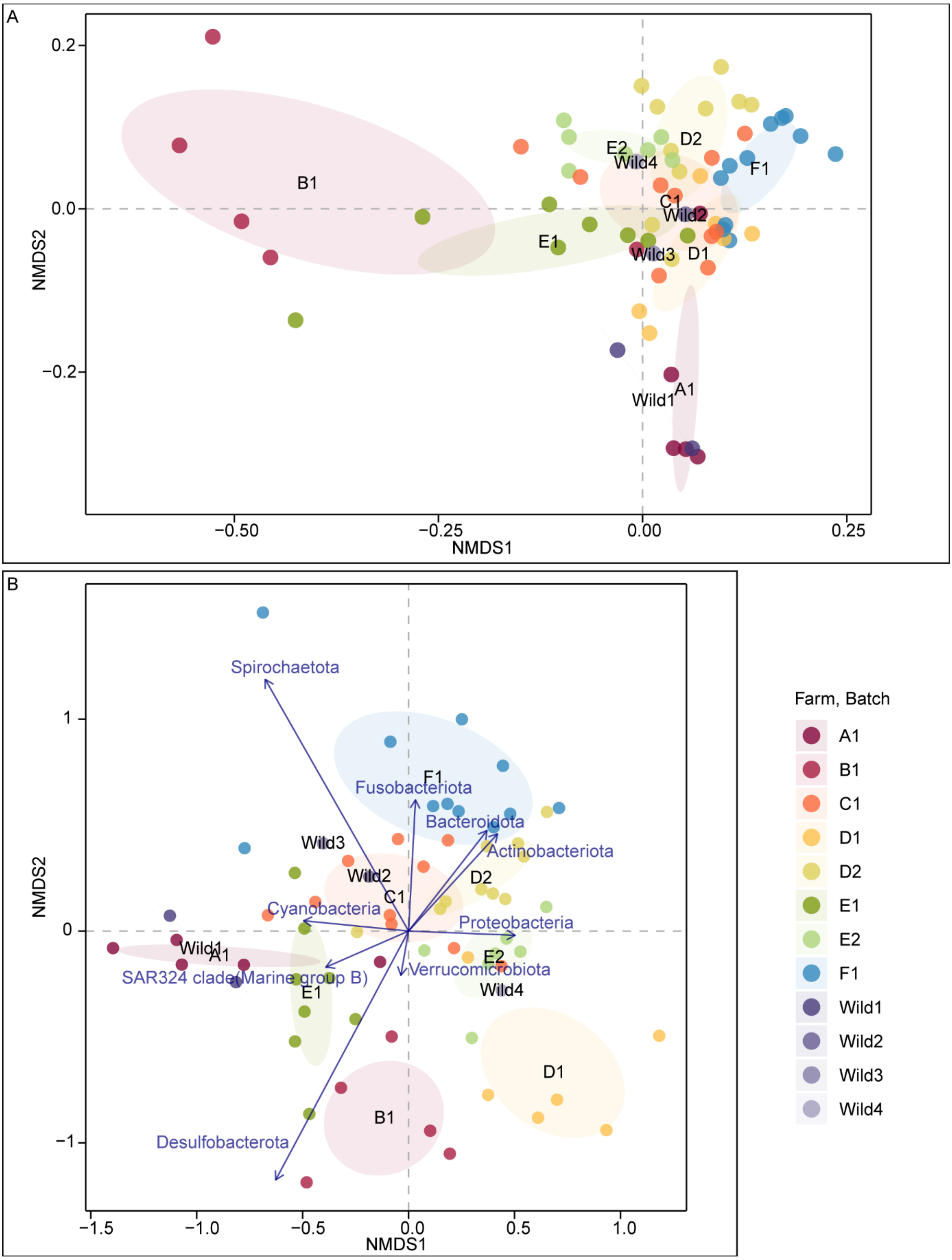

Supplementary Figure 3: Farm and batch effects on gut microbiota β-diversity. Wild and plate-sized farm Asian seabass were included. Gut microbiota analyzed using A) weighted UniFrac

distances and B) Bray-Curtis dissimilarity. All segments from each fish grouped together as one point on the plot. In the legend, letters A to F refer to the six farms where plate-sized seabass were obtained, “wild” refers to the wild seabass, and the subsequent number refers to the batch of seabass. Points and ellipses were colored by sampling farm and batch. Farm and batch text on plot indicates centroid, with ellipses indicating standard deviation. Blue vectors indicate the effect of bacterial phyla observed.

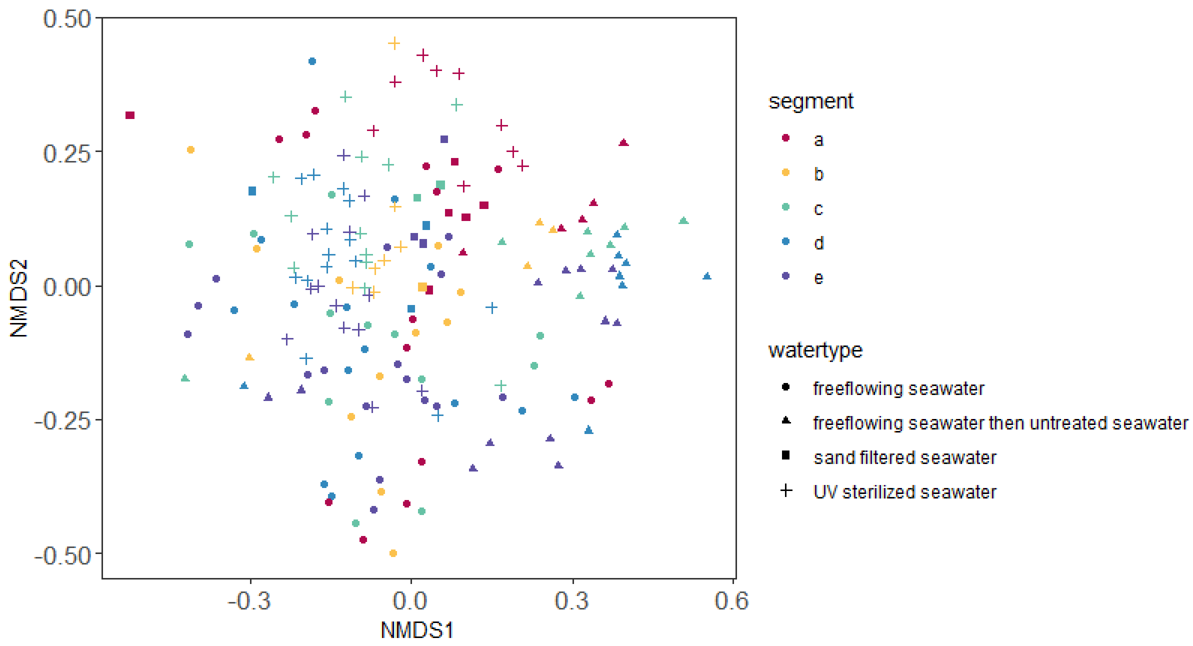

Supplementary Figure 4: β-diversity of plate-sized farmed seabass. NMDS was calculated using Bray-Curtis dissimilarity. Each point represents one sample, coloured based on gut segment and shaped based on fish living conditions.
